# 固相萃取-气相色谱-串联质谱法同时测定武汉市普通人群血清中35种有机氯农药与多氯联苯

**DOI:** 10.3724/SP.J.1123.2021.12013

**Published:** 2022-05-08

**Authors:** Xiang LI, Limei WANG, Lulu SONG, Zhengce WAN, Jing KOU, Mingye ZHANG, Yongman LÜ, Youjie WANG, Surong MEI

**Affiliations:** 1.华中科技大学公共卫生学院环境医学研究所, 教育部环境与健康重点实验室, 湖北 武汉 430030; 1. Key Laboratory of Environment & Health of Ministry of Education, Institute of Environmental Medicine, School of Public Health, Huazhong University of Science and Technology, Wuhan 430030, China; 2.华中科技大学公共卫生学院儿少卫生与妇幼保健系, 湖北 武汉 430030; 2. Department of Maternal and Child Health, School of Public Health, Huazhong University of Science and Technology, Wuhan 430030, China; 3.华中科技大学同济医学院附属同济医院健康管理中心, 湖北 武汉 430030; 3. Health Management Center, Tongji Hospital, Tongji Medical College, Huazhong University of Science and Technology, Wuhan 430030, China

**Keywords:** 气相色谱-串联质谱, 多氯联苯, 有机氯农药, 血清样本, 浓度分布特征, gas chromatography-tandem mass spectrometry (GC-MS/MS), polychlorinated biphenyls (PCBs), organochlorine pesticides (OCPs), serum samples, concentration distribution

## Abstract

有机氯农药(OCPs)和多氯联苯(PCBs)是两类重要的持久性有机污染物,可在环境介质中长期存在,并通过多种途径进入人体,导致人体的高暴露风险。OCPs和PCBs对人体存在诸多健康危害,精准定量人体内OCPs和PCBs的暴露水平是健康效应评价的关键。该研究基于固相萃取-气相色谱-串联质谱联用技术(SPE-GC-MS/MS)建立了同时检测100 μL血清中35种OCPs和PCBs的分析方法。血清样品经尿素沉淀蛋白后,采用Oasis^®^ HLB小柱净化,正己烷-二氯甲烷混合溶液(1∶1, v/v)洗脱,氮吹近干,正己烷定容,多反应监测(MRM)模式检测,内标法定量分析。结果表明,OCPs和PCBs在0.05~50.0 ng/mL范围内线性关系良好,检出限在1.2~71.4 ng/L之间。35种目标分析物的加标回收率在72.6%~142%之间,相对标准偏差小于25%。利用所建立的方法检测了武汉市普通人群血清样本中OCPs和PCBs的浓度水平,结果表明武汉市普通人群广泛暴露于OCPs和PCBs,且以OCPs为主。有8种OCPs和7种PCBs检出率高于50%,其中*p*,*p*'-滴滴伊、*p*,*p*'-滴滴滴和甲氧滴滴涕检出率达100%,非类二噁英PCBs是PCBs的主要成分。血清中OCPs浓度随年龄增长呈升高趋势,在60岁以上存在性别差异;不同性别、年龄人群血清中PCBs浓度无统计学差异。该方法样本用量少,操作简便,具有较高的准确度和精密度,适用于环境健康研究中大量人群血清样本中痕量OCPs和PCBs的生物监测。

## Abstract (en)

Owing to their persistence, ease of accumulation in organisms, and high toxicity, the use of persistent organic pollutants (POPs) has been limited ever since the Stockholm Convention on Persistent Organic Pollutants was signed in 2001 by the United Nations Environment Programme (UNEP). As typical POPs, organochlorine pesticides (OCPs) and polychlorinated biphenyls (PCBs) can persist in the environment for long periods. They can enter human bodies through many pathways and pose a high exposure risk to humans. OCPs and PCBs can lead to endocrine disruption, neurotoxicity, immunotoxicity, reproductive toxicity, and cancer in human beings. Accurate quantification of pollutant load levels *in vivo* is crucial for the evaluation of health effects.

In this study, a rapid and sensitive method based on solid phase extraction-gas chromatography-tandem mass spectrometry (SPE-GC-MS/MS) was developed for the simultaneous determination of 35 OCPs and PCBs in serum. Accordingly, 100 μL of the serum sample was gently mixed with the isotope-labeled internal standard solution (10 μL) to obtain a final mass concentration of 10 ng/mL for each internal standard. After incubation overnight, the samples were mixed with 100 μL purified water for dilution. After protein precipitation with 100 mg urea, the serum samples were passed through preconditioned Oasis^®^ HLB cartridges, washed with 6 mL purified water, and eluted with 5 mL hexane-dichloromethane (1∶1, v/v). The SPE eluant was collected, evaporated to near dryness under a gentle nitrogen stream, and dissolved in 100 μL *n*-hexane. The reconstitution in the vial insert was injected into the GC-MS/MS instrument for analysis. The analytes were separated on an Agilent J&W DB-5MS capillary column (30 m×0.25 mm×0.25 μm) with temperature programming. The mass spectrometer was operated in the electron ionization (EI) mode. The optimal mass spectrometry conditions were realized by optimizing the instrument parameters such as ion pairs and collision energies. The analytes were detected in the multiple reaction monitoring (MRM) mode, and the internal standard method was used for quantitative analysis. The OCPs and PCBs had good linearities in the range of 0.05-50.0 ng/mL. The limits of detection (LODs, *S/N*=3) ranged from 1.2 to 71.4 ng/L. The recoveries of the 35 compounds were 72.6%-142% with relative standard deviations (RSDs) of less than 25% at the three spiked levels. The developed SPE-GC-MS/MS method was successfully applied to the simultaneous analysis of OCPs and PCBs in serum samples obtained from the general population in Wuhan. The results showed that the general population in Wuhan was widely exposed to OCPs and PCBs, especially the former. The detection frequencies of eight OCPs and seven PCBs were greater than 50%, and *p*,*p'*-DDE, *p*,*p'*-DDD, and methoxychlor were detected in all serum sample pools. Non-dioxin-like PCBs (NDL-PCBs) were the dominant PCB congeners, while PCB-28, PCB-153, and PCB-52 were the dominant PCBs in the general population of Wuhan. The concentration of OCPs increased with age. Moreover, the concentration of OCPs in individuals who were more than 66 years old was significantly higher as compared to that in younger individuals. The positive association differing by gender was significant in individuals over 60 years of age. There were no significant differences in PCB concentrations according to gender or age. There were no seasonal differences in the residue levels of OCPs and PCBs in the general population of Wuhan. The developed method is rapid and sensitive; it has the advantages of low limits of detection, satisfactory recoveries, accurate precision, and microsample volume, thus allowing for the simultaneous analysis of trace OCPs and PCBs in microserum samples in epidemiological studies. This robust analytical method also provides a powerful tool for the health risk assessment of OCP and PCB exposure.

有机氯农药(organochlorine pesticides, OCPs)和多氯联苯(polychlorinated biphenyls, PCBs)是联合国环境规划署签署的“斯德哥尔摩公约”中两类主要的持久性有机污染物(persistent organic pollutants, POPs),具有高毒性、持久性、生物蓄积性和远距离迁移性^[1]^。我国于20世纪70~80年代陆续禁产禁用OCPs和PCBs,但OCPs和PCBs仍可以在自然水体、土壤、食品及大气中广泛检出^[2⇓⇓-5]^。环境介质及食品中OCPs、PCBs的普遍污染导致人群的高暴露风险,对人类造成潜在的健康危害,如内分泌干扰效应、神经毒性、免疫毒性、生殖毒性和致癌效应等^[6⇓⇓-9]^。因此,人体内OCPs与PCBs暴露水平与健康风险的评估至关重要。

建立灵敏快速的人体内OCPs和PCBs的检测方法可为环境暴露与健康风险评估提供技术支持。高脂溶性的OCPs和PCBs易蓄积于人体的脂肪组织,但在实际研究中,脂肪组织不易获取^[10]^。在平衡状态下,血液中POPs的浓度可以代表蓄积在脂肪组织中的POPs浓度^[11,12]^;且血液获取方便快捷,易获得不同年龄层的样本,因此血液被广泛用作评价OCPs、PCBs在人体内暴露水平的生物监测样本^[13⇓-15]^。人体血液成分复杂,生物体内残留的OCPs、PCBs多处于痕量甚至超痕量水平,高效的前处理方法既可以实现对目标物的高效富集,又可以降低或去除基质的干扰^[16]^。相较于传统的液液萃取技术(liquid-liquid extraction, LLE),固相萃取技术(solid phase extraction, SPE)溶剂使用量少,样品富集与净化可以同时完成,使得检测灵敏度大幅提高^[17]^。OCPs和PCBs的仪器分析方法包括气相色谱-电子捕获检测法(GC-ECD)、气相色谱-质谱检测法(GC-MS)、气相色谱-串联质谱检测法(GC-MS/MS)等,其中GC-MS/MS可高效分离多种组分,特异性强、灵敏度高,适用于血液中痕量OCPs和PCBs的精准定量分析^[18⇓-20]^。

目前,国内外已有许多人体内OCPs和PCBs残留的检测研究,屈伟月^[21]^建立的LLE串联硅胶柱-GC-MS/MS检测血清中12种OCPs和43种PCBs的分析方法回收率良好(79%~84%),检测组分多,但前处理操作复杂,样本用量大,且OCPs与PCBs不能同时检测;Stubleski等^[22]^建立的96孔板固相萃取-GC-MS/MS检测血中5种OCPs和16种PCBs的分析方法样本用量少,检出限在2.2~167.0 ng/L之间,但回收率不甚理想(31%~63%)。

基于此,本研究建立了一种快速简便、样本用量少、回收率和精密度良好的SPE-GC-MS/MS同时检测人血清中35种OCPs和PCBs的分析方法,并应用于武汉市普通人群体内OCPs和PCBs的浓度分布特征研究,为进一步开展人群健康效应研究提供技术支撑和基础数据。

## 1 实验部分

### 1.1 仪器、试剂与材料

Trace 1300气相色谱-TSQ 8000三重四极杆质谱仪(美国Thermo Fisher公司);氮吹仪(赛多利斯科学仪器(北京)有限公司);固相萃取仪(美国Agilent公司);隔膜真空泵(美国GAST公司)。

Oasis^®^ HLB固相萃取柱(1 mL/30 mg,美国Waters公司),胎牛血清(美国Thermo Fisher公司),尿素(分析纯,中国国药集团),二氯甲烷(农残级,美国J. T. Baker公司),甲醇(色谱纯,德国默克集团),正己烷(农残级,美国Sigma-Aldrich公司),纯净水(杭州娃哈哈集团)。

17种OCPs混合标准溶液(含*α*-六六六(*α*-HCH)、*β*-六六六(*β*-HCH)、*γ*-六六六(*γ*-HCH)、*δ*-六六六(*δ*-HCH)、*p*,*p*'-滴滴伊(*p*,*p*'-DDE)、*p*,*p*'-滴滴滴(*p*,*p*'-DDD)、*p*,*p*'-滴滴涕(*p*,*p*'-DDT)、艾氏剂(aldrin)、狄氏剂(diedrin)、异狄氏剂(endrin)、异狄氏剂醛(endrin aldehyde)、*α*-氯丹(*α*-chlordane)、*γ*-氯丹(*γ*-chlordane)、硫丹Ⅱ(endosulfan Ⅱ)、硫丹硫酸盐(endosulfan sulfate)、环氧七氯(heptachlor epoxide)、甲氧滴滴涕(methoxychlor))和18种PCBs混合标准溶液(含PCB-28、PCB-52、PCB-77、PCB-81、PCB-101、PCB-105、PCB-114、PCB-118、PCB-123、PCB-126、PCB-138、PCB-153、PCB-156、PCB-157、PCB-167、PCB-169、PCB-180、PCB-189)均购自美国AccuStandard公司。4种OCPs同位素内标^13^C_6_-*γ*-HCH、^13^C_12_-*p*,*p*'-DDE、^13^C_12_-*p*,*p*'-DDD、^13^C_12_-*p*,*p*'-DDT和18种^13^C_12_-PCBs同位素内标均购自美国CIL公司。

### 1.2 标准溶液的配制

混合标准贮备液:用正己烷稀释17种OCPs和18种PCBs混合标准溶液,配制成1 μg/mL的35种OCPs和PCBs混合标准贮备液,于-20 ℃保存。

标准工作溶液:准确量取一定量混合标准贮备液,用正己烷稀释,配制成50、20、10、5、2、1、0.50、0.20、0.10 ng/mL的系列标准溶液。

### 1.3 样品前处理

每份血清样本取100 μL,加入10 μL质量浓度为100 ng/mL的同位素内标溶液,涡旋混匀后置于4 ℃过夜;取出样本,用100 μL纯净水稀释,加入100 mg尿素,涡旋混匀,超声25 min;依次用3 mL二氯甲烷、5 mL甲醇、5 mL纯净水活化平衡Oasis^®^ HLB小柱;将样品全部过柱,并用1 mL纯净水润洗样品管两次,将液体全部导入HLB小柱,流速0.5~1 mL/min;用6 mL纯净水淋洗HLB小柱,真空抽干45 min;用5 mL正己烷-二氯甲烷混合溶液(1∶1, v/v)洗脱,收集全部洗脱液。洗脱液氮吹近干,用正己烷定容至100 μL,进样测定。

### 1.4 分析条件

色谱条件:DB-5MS毛细管色谱柱(30 m×0.25 mm×0.25 μm,美国Agilent公司);以高纯氦气(99.999%)作为载气,柱流量1.0 mL/min,恒流模式;不分流进样模式,进样量1 μL;进样口温度250 ℃。色谱柱升温程序:初始温度60 ℃,保持1 min,以30 ℃/min升至180 ℃,保持1 min,以3 ℃/min升至280 ℃,保持5 min。

质谱条件:三重四极杆质谱仪,配备电子轰击离子源,电离电压70 eV;离子源温度230 ℃,四极杆温度150 ℃;定量分析采用多反应监测(MRM)模式,内标法定量。

### 1.5 血清样本采集

2018~2019年在武汉市同济医院体检中心采集武汉市普通人群血清样本共4132份,依据采样时间(2018年秋季、2019年春季)、年龄(10个年龄组:≤30岁;31~35岁;36~40岁;41~45岁;46~50岁;51~55岁;56~60岁;61~65岁;66~70岁;>70岁)和性别将血清样本混合为40个样本池。本研究得到华中科技大学同济医学院伦理委员会批准,招募志愿者均签署知情同意书。

## 2 结果与讨论

### 2.1 质谱条件的优化

按1.4节分析条件建立全扫描方法,根据化合物信息确定母离子后运行子离子扫描,选择丰度最高的两对离子对作为目标化合物的定量离子对和定性离子对,并确定每对离子对的最佳碰撞能量。优化后各目标物和内标的质谱分析参数见表1。

**表1:** 35种目标物与22种内标的质谱分析参数

Analyte	*t*_R_/ min	Quantitative analysis			Qualitative analysis			Analyte	*t*_R_/ min	Quantitative analysis			Qualitative analysis		
		Ion pair (*m/z*)	CE/ eV		Ion pair (*m/z*)	CE/ eV				Ion pair (*m/z*)	CE/ eV		Ion pair (*m/z*)		CE/ eV
*α*-HCH	9.47	219/183	10		219/147	25		*p*,*p*'-DDD	21.06	235/165	15		235/199		15
*β*-HCH	10.14	181/145	15		219/183	10		^13^C_12_-PCB-114	21.06	338/268	25		338/303		15
^13^C_6_-*γ*-HCH	10.41	189/154	15		189/133	10		PCB-114	21.07	326/256	25		326/291		15
*γ*-HCH	10.43	181/145	15		219/183	10		Endrin aldehyde	21.42	250/215	25		345/246		25
*δ*-HCH	11.29	219/183	10		181/145	15		^13^C_12_-PCB-153	21.69	372/302	25		372/337		10
^13^C_12_-PCB-28	12.39	268/198	25		268/233	10		PCB-153	21.71	360/290	32		360/325		15
PCB-28	12.40	256/186	25		256/151	60		^13^C_12_-PCB-105	21.83	338/268	25		338/303		15
^13^C_12_-PCB-52	13.70	304/233	25		304/269	10		PCB-105	21.85	326/256	25		326/291		15
PCB-52	13.72	292/222	30		292/257	15		Endosulfan sulfate	22.68	272/237	15		272/141		35
Aldrin	14.37	263/193	30		263/228	20		^13^C_12_-*p*,*p*'-DDT	23.06	247/177	20		247/211		15
Heptachlor epoxide	16.05	353/263	15		353/282	15		*p*,*p*'-DDT	23.07	235/165	15		235/200		10
*α*-Chlordane	17.14	373/266	20		373/301	10		^13^C_12_-PCB-138	23.07	372/302	25		372/337		10
^13^C_12_-PCB-101	17.50	338/268	25		338/303	10		PCB-138	23.07	360/290	30		360/325		15
PCB-101	17.51	326/256	30		326/291	15		^13^C_12_-PCB-126	23.61	338/268	25		338/303		15
*γ*-Chlordane	17.76	373/266	20		373/301	10		PCB-126	23.62	326/256	25		326/291		15
^13^C_12_-PCB-81	18.81	304/233	25		304/268	15		^13^C_12_-PCB-167	24.63	372/302	25		372/337		15
PCB-81	18.83	292/220	25		292/257	15		PCB-167	24.65	360/290	25		360/325		15
^13^C_12_-*p*,*p*'-DDE	18.93	258/189	30		258/222	20		^13^C_12_-PCB-156	25.77	372/302	25		372/337		15
*p*,*p*'-DDE	18.93	246/176	20		246/211	20		PCB-156	25.79	360/290	25		360/325		15
Dieldrin	19.07	277/241	10		277/206	15		^13^C_12_-PCB-157	26.02	372/302	25		372/337		15
^13^C_12_-PCB-77	19.32	304/234	25		304/269	15		PCB-157	26.04	360/290	25		360/325		15
PCB-77	19.33	292/220	25		292/257	15		Methoxychlor	26.25	227/169	20		227/184		20
Endrin	20.09	281/245	10		281/173	40		^13^C_12_-PCB-180	26.67	406/336	25		406/371		10
^13^C_12_-PCB-123	20.38	338/268	25		338/303	15		PCB-180	26.69	394/324	32		394/359		15
PCB-123	20.38	326/256	25		326/291	15		^13^C_12_-PCB-169	27.84	372/302	25		372/337		15
^13^C_12_-PCB-118	20.59	338/268	25		338/303	15		PCB-169	27.89	360/290	25		360/325		15
PCB-118	20.59	326/256	30		326/291	15		^13^C_12_-PCB-189	29.78	406/336	25		406/371		15
Endosulfan Ⅱ	20.66	195/159	10		241/206	15		PCB-189	30.05	394/324	25		394/359		15
^13^C_12_-*p*,*p*'-DDD	21.05	247/177	20		247/211	15									

*t*_R_: retention time; CE: collision energy; HCH: hexachlorocyclohexane.

### 2.2 方法学评价

#### 2.2.1 线性范围、方法检出限与定量限

按1.3节前处理流程处理空白胎牛血清之后,向其中加入不同浓度的混合标准溶液,配制质量浓度为0.01~50.0 ng/mL的基质匹配标准溶液,采用上述分析条件,以内标法进行定量分析,目标物标准曲线的线性范围、相关系数(*r*^2^)及方法检出限(LOD)和定量限(LOQ)见表2。35种OCPs和PCBs在0.05~50.0 ng/mL范围内线性关系良好,相关系数在0.989~0.999之间;以3倍信噪比相对应的浓度作为方法的检出限,以10倍信噪比确定方法的定量限,35种OCPs和PCBs的检出限在1.2~71.4 ng/L之间,定量限在4.1~238.1 ng/L之间。

**表2:** 35种分析物的线性范围、相关系数、方法检出限和定量限

Analyte	Linear range/ (ng/mL)	*r*^2^	LOD/ (ng/L)	LOQ/ (ng/L)	Analyte	Linear range/ (ng/mL)	*r*^2^	LOD/ (ng/L)	LOQ/ (ng/L)	
*α*-HCH	0.05-50	0.999	10.2	33.9	PCB-52	0.02-50	0.999	5.0	16.5	
*β*-HCH	0.10-50	0.996	15.2	50.7	PCB-77	0.05-50	0.999	9.7	32.3	
*γ*-HCH	0.50-50	0.999	71.4	238.1	PCB-81	0.05-50	0.999	6.8	22.7	
*δ*-HCH	0.01-50	0.996	2.2	6.7	PCB-101	0.10-50	0.998	25.6	83.3	
*p*,*p*'-DDE	0.02-50	0.999	5.6	18.5	PCB-105	0.05-50	0.999	9.3	29.7	
*p*,*p*'-DDD	0.01-50	0.999	2.9	9.6	PCB-114	0.05-50	0.999	12.1	40.3	
*p*,*p*'-DDT	0.05-50	0.999	9.1	30.4	PCB-118	0.05-50	0.999	7.9	26.4	
Aldrin	0.10-50	0.999	20.0	67.0	PCB-123	0.10-50	0.999	9.1	30.5	
Diedrin	0.05-50	0.999	12.8	42.6	PCB-126	0.05-50	0.999	10.2	33.8	
Endrin	0.10-50	0.998	24.5	81.6	PCB-138	0.02-50	0.999	5.1	16.8	
Endrin aldehyde	0.10-50	0.989	21.7	72.2	PCB-153	0.10-50	0.999	16.9	56.5	
*α*-Chlordane	0.05-50	0.999	14.6	49.6	PCB-156	0.10-50	0.994	20.0	63.9	
*γ*-Chlordane	0.05-50	0.997	6.8	23.9	PCB-157	0.05-50	0.999	8.1	25.9	
Endosulfan Ⅱ	0.05-50	0.998	6.9	23.0	PCB-167	0.20-50	0.999	39.1	132.1	
Endosulfan sulfate	0.05-50	0.998	12.5	41.7	PCB-169	0.05-50	0.999	10.0	31.8	
Heptachlor epoxide	0.01-50	0.999	20.0	78.1	PCB-180	0.05-50	0.996	9.2	28.9	
Methoxychlor	0.01-50	0.999	1.2	4.1	PCB-189	0.05-50	0.999	10.0	34.8	
PCB-28	0.05-50	0.999	8.8	29.1						

#### 2.2.2 方法的准确度与精密度

采用胎牛血清进行加标回收率试验,分别向胎牛血清中添加不同浓度的混合标准溶液,每一组浓度做6个平行样,计算加标回收率和相对标准偏差(RSD)。在检测样本的同时检测空白胎牛血清和超纯水基质,监测仪器性能是否稳定并控制实验过程中可能存在的污染情况。35种OCPs和PCBs的加标回收率在72.6%~142%之间,RSD小于25%(见表3)。

**表3:** 35种分析物在胎牛血清中3个水平下的加标回收率和精密度(*n*=6)

Analyte	5 ng/mL			10 ng/mL			50 ng/mL		Analyte	5 ng/mL			10 ng/mL			50 ng/mL		
	Recovery/ %	RSD/ %		Recovery/ %	RSD/ %		Recovery/ %	RSD/ %		Recovery/ %	RSD/ %		Recovery/ %	RSD/ %		Recovery/ %		RSD/ %
*α*-HCH	121	9.7		93.7	17		117	17	PCB-52	95.9	6.0		83.1	4.3		89.5		2.9
*β*-HCH	121	15		108	25		133	13	PCB-77	95.3	6.8		82.1	5.3		90.1		5.3
*γ*-HCH	114	11		92.9	16		116	22	PCB-81	98.9	6.3		84.2	8.4		91.2		3.7
*δ*-HCH	118	11		103	23		125	11	PCB-101	100	6.6		86.7	2.2		95.1		6.6
*p*,*p*'-DDE	106	9.6		87.1	3.8		95	3.5	PCB-105	98.0	7.8		82.8	5.4		95.8		5.7
*p*,*p*'-DDD	93.0	4.6		80.9	6.5		93	3.4	PCB-114	102	11		93.3	7.5		105		5.6
*p*,*p*'-DDT	99.2	10		88.1	6.8		103	6.7	PCB-118	92.6	7.0		80.1	4.9		89.8		5.7
Aldrin	87.1	12		76.8	5.7		79.2	17	PCB-123	98.8	7.4		82.8	8.5		90.7		6.3
Diedrin	127	12		88.4	20		91.9	12	PCB-126	98.1	9.1		91.4	3.7		99.0		4.4
Endrin	111	4.3		92.2	8.2		89.2	14	PCB-138	92.6	7.4		81.2	4.1		100		3.4
Endrin aldehyde	136	15		134	21		130	21	PCB-153	93.9	6.2		86.4	8.5		93.4		6.9
*α*-Chlordane	94.6	6.9		83.3	6.0		92.3	14	PCB-156	101	3.1		90.1	8.3		108		6.8
*γ*-Chlordane	105	7.5		90.8	8.1		99.9	12	PCB-157	99.2	16		83.4	7.4		90.9		8.1
Endosulfan Ⅱ	142	21		103	19		120	21	PCB-167	87.2	11		72.6	10		82.4		8.0
Endosulfan sulfate	124	12		100	18		129	20	PCB-169	88.6	13		83.4	8.3		97.8		9.3
Heptachlor epoxide	105	2.9		84.1	4.1		97.0	13	PCB-180	94.0	14		88.7	7.8		98.9		8.4
Methoxychlor	125	8.6		101	15		124	13	PCB-189	108	13		92.6	5.1		104		3.3
PCB-28	99.5	7.6		82.4	6.6		90.6	2.1										

### 2.3 方法学比较

血清样本珍贵,不易获取,其他文献报道的检测方法的样本使用量一般不少于500 μL^[23⇓⇓-26]^,本研究检测血清用量只需要100 μL。Wittsiepe等^[27]^和Stubleski等^[22]^的方法样本使用量少,分别为200 μL和150 μL,但检测的OCPs组分少,加标回收率不甚理想(见表4),本研究检测组分多,检出限低,准确度与精密度高。同时,本方法操作简便,经过固相萃取就能有效去除杂质和富集痕量待测组分,简化了繁琐的前处理步骤,在大样本人群监测中能节约大量人力物力。

**表4:** 本方法与其他文献方法的比较

Analytes	Matrix	Sample volume/μL	LODs/ (ng/L)		Recoveries/ %		RSDs/%	Analytical methods	Reference	
17 OCPs, 18 PCBs	serum	100	1.2-	71.4	72.6-	141.9	<24.6	SPE-GC-MS/MS	this work	
7 OCPs, 6 PCBs	serum	500	60.0-	360.0	55-	115	2.0-14.6	GPC-GC-ECD	[23]	
15 OCPs, 20 PCBs	serum	3000	10.0-	500.0	70-	97	<10	LLE-GC-ECD	[24]	
11 OCPs, 15 PCBs	plasma	500	5.0-	700.0	-		<20.8	SPE-GC-MS	[25]	
5 OCPs, 16 PCBs	plasma	500	8.0-	117.7	46-	110	<25	SPE-GC-HRMS	[26]	
5 OCPs, 20 PCBs	serum	200	3.0-	28.0	25.7-	114.4	5-11	SPE-GC-HRMS	[27]	
5 OCPs, 16 PCBs	serum/plasma	150	2.2-	167.0	31-	63	11-25	96-well plate SPE-GC-MS/MS	[22]	

GPC: gel permeation chromatography; HRMS: high resolution mass spectrometry.

### 2.4 武汉市普通人群体内OCPs和PCBs的浓度分布特征研究

采用所建立的方法检测血清样本池中35种OCPs和PCBs浓度,从表5的结果发现:①武汉市普通人群血清中OCPs和PCBs的污染普遍存在,且以OCPs为主,其浓度水平依次为:*p*,*p*'-滴滴伊>*β*-六六六>*γ*-六六六>*p*,*p*'-滴滴涕>狄氏剂,其中*p*,*p*'-滴滴伊、*p*,*p*'-滴滴滴和甲氧滴滴涕检出率达100%; ②本研究武汉市普通人群中OCPs的浓度水平与北京市普通人群暴露水平一致,低于江苏普通人群OCPs暴露水平^[28,29]^。*p*,*p*'-滴滴伊/*p*,*p*'-滴滴涕比值通常作为*p*,*p*'-滴滴涕历史累积的指标,该比值小于10表明*p*,*p*'-滴滴伊主要来源于滴滴涕的近期暴露,比值大于10表明*p*,*p*'-滴滴伊来源于滴滴涕的历史暴露^[30]^,本研究中*p*,*p*'-滴滴伊/*p*,*p*'-滴滴涕比值为58,推断武汉市普通人群血清中*p*,*p*'-滴滴伊的蓄积可能主要来自于滴滴涕的历史残留; ③从PCBs同系物组成来看,武汉市普通人群血清中主要以PCB-28、PCB-153和PCB-52为主,非类二噁英PCBs是主要成分,与国内其他人群体内PCBs的组成特征基本一致^[31]^。

**表5:** 血清样本中OCPs和PCBs的质量浓度(*n*=40)

Analyte	Detection frequency/ %	Mean±SD/ (ng/mL)	Median/ (ng/mL)	Range/ (ng/mL)	Analyte	Detection frequency/ %	Mean±SD/ (ng/mL)	Median/ (ng/mL)	Range/ (ng/mL)
*α*-HCH	67.5	0.023±0.005	0.146	<LOD-1.025	PCB-52	87.5	0.038±0.035	0.023	< LOD-0.135
*β*-HCH	97.5	0.379±0.098	0.147	<LOD-3.435	PCB-77	15.0	<LOD	<LOD	<LOD-0.024
*γ*-HCH	7.5	0.193±0.247	<LOD	<LOD-1.107	PCB-81	52.5	0.010±0.006	0.008	<LOD-0.027
*δ*-HCH	42.5	0.0020±0.0004	<LOD	<LOD-0.011	PCB-101	10.0	<LOD	<LOD	<LOD-0.053
*p*,*p*'-DDE	100.0	8.673±1.219	5.793	1.496-37.503	PCB-105	25.0	<LOD	<LOD	<LOD-0.024
*p*,*p*'-DDD	100.0	0.099±0.005	0.095	0.014-0.186	PCB-114	22.5	<LOD	<LOD	<LOD-0.076
*p*,*p*'-DDT	70.0	0.149±0.184	0.073	<LOD-0.910	PCB-118	87.5	0.034±0.023	0.026	< LOD-0.089
Aldrin	30.0	0.037±0.059	<LOD	<LOD-0.189	PCB-123	25.0	<LOD	<LOD	<LOD-0.022
Heptachlor epoxide	2.5	<LOD	<LOD	<LOD-0.024	PCB-126	15.0	<LOD	<LOD	<LOD-0.038
*α*-Chlordane	15.0	<LOD	<LOD	<LOD-0.055	PCB-138	67.5	0.031±0.035	0.017	<LOD-0.114
*γ*-Chlordane	25.0	<LOD	<LOD	<LOD-0.054	PCB-153	75.0	0.069±0.055	0.056	< LOD-0.212
Diedrin	82.5	0.148±0.120	0.137	<LOD-0.507	PCB-156	0.0	<LOD	<LOD	< LOD
Endrin	45.0	0.030±0.028	<LOD	<LOD-0.096	PCB-157	12.5	<LOD	<LOD	<LOD-0.032
Endosulfan Ⅱ	72.5	0.078±0.079	0.061	<LOD-0.303	PCB-167	10.0	<LOD	<LOD	<LOD-0.085
Endrin aldehyde	25.0	0.010±0.060	<LOD	<LOD-0.207	PCB-169	7.5	<LOD	<LOD	<LOD-0.018
Endosulfan sulfate	15.0	0.010±0.008	<LOD	<LOD-0.040	PCB-180	52.5	0.022±0.030	0.010	<LOD-0.114
Methoxychlor	100.0	0.032±0.023	0.030	0.005-0.099	PCB-189	5.0	<LOD	<LOD	<LOD-0.017
PCB-28	77.5	0.126±0.127	0.099	<LOD-0.593					

SD: standard deviation; <LOD: below the limit of detection.

### 2.5 血清中OCPs和PCBs浓度水平与年龄、性别的关系

血清中OCPs浓度随年龄增加呈升高趋势(见图1a), 66岁以上人群血清中OCPs浓度显著高于66岁以下人群(*p*<0.05),与我国莱州湾地区、吉林伊通等地人群血清样本池中OCPs浓度变化趋势一致,这可能是由于随着时间推移,经由各种途径暴露的OCPs在人体中出现累积效应,提示年龄是影响人体OCPs负荷水平的重要因素^[32,33]^。46~55岁以及61~65岁人群血清中PCBs浓度水平略高于其他年龄组,但无显著性差异(见图1b),与Meng等^[34]^的研究结果一致,这可能是因为虽然我国从20世纪80年代开始禁止生产使用PCBs,但工业焚烧和废旧电器的泄漏使得我国居民处于PCBs的持续暴露中^[35,36]^。

**图1:**
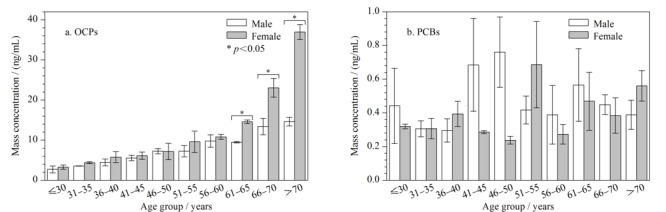
不同年龄、性别血清样本池中OCPs和PCBs的浓度分布(*n*=40)

60岁以下女性血清中OCPs浓度水平和相同年龄段男性血清中OCPs浓度水平无显著性差异,而60岁以上女性血清中OCPs浓度水平较高,是相同年龄段男性血清中OCPs浓度的2~3倍(*p*<0.05,见图1a),与Thomas等^[37]^、Porta等^[38]^的研究结果基本一致,这可能与男女饮食习惯的差异有关。与男性相比,女性偏向于摄入更多的蔬菜水果,而蔬菜水果中往往含有较高的OCPs残留^[39]^。男性和女性血清中PCBs浓度水平无统计学差异(见图1b),与Zheng等^[40]^、Du等^[41]^的研究结果一致。不同季节(2018年秋季、2019年春季)收集的血清样本池OCPs和PCBs浓度水平也无统计学差异,可能是因为春秋两季人群膳食模式和食物来源无明显变化。

## 3 结论

本研究建立了固相萃取-气相色谱-串联质谱联用技术同时检测人血清中35种OCPs和PCBs的方法,该方法检出限低、回收率及精密度良好,且样本用量少,操作简便,检测组分多,适用于环境健康研究中大样本人群的生物监测,为OCPs和PCBs的人体内暴露水平与健康风险评估提供技术支持。利用建立的方法检测了武汉市普通人群血清样本中OCPs和PCBs的浓度水平,武汉市普通人群广泛暴露于OCPs和PCBs,且以OCPs为主,非类二噁英PCBs是PCBs的主要成分。血清中OCPs浓度随年龄增长呈升高趋势,OCPs浓度在60岁以上存在性别差异,不同性别血清中PCBs浓度无统计学差异。
